# Computer Simulation-Guided Rational Design of Sulfadiazine-Imprinted Polymers for High-Efficiency Adsorption of Antibiotics in Complex Aquatic Matrices

**DOI:** 10.3390/membranes16040118

**Published:** 2026-03-28

**Authors:** Mengfan Xu, Yanhong Wang, Mingfen Niu, Qiang Zhou, Wang Yang

**Affiliations:** 1 Institute of Applied Ecology, Chinese Academy of Sciences, Shenyang 110016, China; xumengfan5@163.com (M.X.); wangyh@iae.ac.cn (Y.W.); yangw@iae.ac.cn (W.Y.); 2 School of Municipal and Environmental Engineering, Shenyang Jianzhu University, Shenyang 110168, China

**Keywords:** SDZ, DFT calculations, molecularly imprinted polymers, adsorption

## Abstract

To address the limited selectivity of conventional membrane materials toward sulfonamide antibiotics, this study employed a DFT calculation approach to optimize the design of a molecularly imprinted system for sulfadiazine (SDZ). A hierarchical set of template molecules—aniline (ANL), sulfanilamide (SNM), and SDZ—was introduced to systematically elucidate structure-dependent template–monomer matching mechanisms in sulfonamide imprinting systems. Through rational screening, trifluoroethyl methacrylate (TFEMAA) was identified as the optimal functional monomer, with an optimal imprinting molar ratio of 1:4 (SDZ to TFEMAA). Guided by the simulation results, SDZ molecularly imprinted polymers (MIPs) were synthesized via precipitation polymerization and systematically characterized for their morphology and recognition properties. The MIPs exhibited a well-defined spherical morphology with abundant imprinted cavities, achieving adsorption equilibrium within 1.5 h. The adsorption kinetics followed a pseudo-second-order model, indicating a chemisorption-dominated process. Scatchard analysis revealed the presence of both high- and low-affinity binding sites in the MIPs. Selectivity experiments, quantified by distribution coefficients (K_d_) and selectivity coefficients (k), demonstrated a significantly higher adsorption capacity for SDZ than for structural analogs and non-analogs. In real water samples, the MIPs outperformed conventional HLB sorbents and showed strong anti-interference capability (RSD < 3%). This work provides a material foundation for developing highly selective SDZ-imprinted membranes and advances the application of molecular imprinting technology in membrane separation systems.

## 1. Introduction

Sulfadiazine (SDZ), a representative sulfonamide antibiotic, is extensively employed in veterinary husbandry and clinical medicine [1]. Consequently, it has been increasingly detected as a persistent micropollutant in diverse aquatic environments [2]. Its widespread use raises significant concerns: residues in animal-derived products can accumulate in humans through the food chain, posing health risks, while excreta from treated livestock introduce SDZ into ecosystems, resulting in potential ecotoxicological contamination of soil and water bodies [3]. Current remediation technologies face distinct limitations. Conventional biological treatments are often compromised by co-existing pollutants (e.g., microplastics), impairing SDZ removal efficiency [4]. Physical adsorption methods, including those employing biomass or novel materials, are frequently hampered by variable water matrices—specifically ionic strength, pH, and dissolved organic matter—leading to insufficient selectivity for SDZ [5,6]. Although membrane separation offers advantages in efficiency and energy consumption, it struggles with low selectivity and severe membrane fouling when treating antibiotic micropollutants.

Molecular imprinting technology (MIT) presents a promising avenue to enhance the selective recognition capabilities of membrane materials towards sulfonamide antibiotics. This technique creates specific binding sites by copolymerizing template molecules with functional monomers, yielding molecularly imprinted polymers (MIPs) capable of high-selectivity adsorption of target pollutants [7]. While SDZ-based MIPs have found broad application in analytical detection [8,9,10], their potential in membrane separation remains underexplored. For instance, Li et al. [11] fabricated a baicalin-imprinted membrane using deep eutectic solvents and electrospinning, with lignin-cellulose nanofibers as the substrate, achieving an adsorption capacity of 142.1 mg·g^−1^ and maintaining 96.4% efficiency after 10 cycles, demonstrating the green and sustainable potential of biomass-based imprinted membranes. Similarly, Ma et al. [12] constructed a selective berberine separation layer via surface molecular imprinting on a chitosan/polyvinyl alcohol composite membrane, which reached an adsorption capacity of 74.56 mg·g^−1^ within 2 h and exhibited excellent permeation selectivity. These studies highlight that integrating MIPs with membrane substrates can yield composite membranes with both high selectivity and flux, thus emerging as a research hotspot. However, a critical challenge persists in SDZ-MIP fabrication: the conventional trial-and-error approach for screening functional monomers is not only time-consuming and solvent-intensive [13] but also fails to systematically optimize the synergy between imprinting site affinity and adsorption kinetics.

The rapid development of quantum chemistry offers a powerful solution. DFT calculations and quantum chemical analysis of MIP pre-polymerization systems are increasingly employed to efficiently screen functional monomers, cross-linkers, solvents, and optimal imprinting ratios [14]. Examples include Liu et al. [15], who used density functional theory (DFT) calculations to select methyl methacrylate as the optimal monomer for vitexin imprinting at a 1:6 template-to-monomer ratio, and Huang et al. [16], who identified methacrylamide via Gaussian software simulations for optimal imprinting of ginkgolide B at a 1:4 ratio. Somandi et al. [17] systematically evaluated interaction energies between an Autoinducer-2 analog and seven monomers using molecular docking and mechanics, predicting optimal hydrogen-bonding patterns. These studies confirm the maturity of simulations for elucidating imprinting mechanisms and screening parameters. Nevertheless, the current literature lacks a systematic investigation into the matching rules between template molecular structure and monomer functionality, as well as the underlying imprinting mechanisms, leading to inconsistent and often suboptimal template-to-monomer ratios in practice.

**Figure 1:**
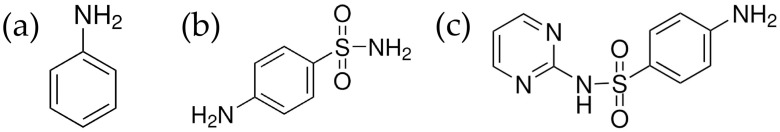
Chemical structures of (**a**) ANL, (**b**) SNM, and (**c**) SDZ.

Building upon our prior work, which successfully demonstrated a computer simulation-guided strategy for developing dibutyl phthalate-imprinted ultrafiltration membranes with notable energy-saving and selective advantages [18], this study further integrates DFT calculations with experimental validation. We aim to systematically screen functional monomers and elucidate the template–monomer matching mechanism for SDZ imprinting. Three templates with increasing structural complexity were selected: aniline (ANL), sulfanilamide (SNM, the core scaffold of sulfonamides), and SDZ (containing a bicyclic pyrimidine ring) (Figure 1). This hierarchical template design was introduced to elucidate the structure-dependent matching mechanism between sulfonamide scaffolds and functional monomers, rather than to replace SDZ as the final imprinting target. Specifically, ANL was used as a simplified aromatic amine model. SNM represented the fundamental sulfonamide scaffold, and SDZ served as the actual target molecule for experimental imprinting. Six functional monomers from three categories were evaluated: (1) carboxylic acids (MAA and TFMAA); (2) acrylates (EMA and MMA); and (3) N-heterocycles (4-HP and NVP). Guided by simulation results, SDZ-MIPs were synthesized via precipitation polymerization. Based on characterization by SEM and FT-IR, a regular spherical morphology with effective imprinting cavities was confirmed. Adsorption studies showed equilibrium within 1.5 h, and Scatchard analysis revealed the presence of both high- and low-affinity binding sites. The MIPs exhibited superior selectivity for SDZ over structural analogs and outperformed commercial HLB sorbents in real water samples with strong anti-interference capability. This work not only establishes a theoretical and technical paradigm for the deep remediation of SDZ in complex water matrices but also provides a material basis for future membrane-oriented applications. The integration of these MIPs into continuous membrane separation systems will require further immobilization strategies and continuous-flow evaluation in future studies.

## 2. Materials and Method

### 2.1. Reagents and Instruments

Sulfadiazine (SDZ, 98%), sulfanilamide (SNM, 98%), sulfamethoxazole (SMX, 98%), and sulfometuron-methyl (SMM, 98%) were purchased from Shandong Keyuan Biochemical Co., Ltd. (Jinan, Shandong, China). 2,2′-Azobis(2-methylpropionitrile) (AIBN) was obtained from Tianjin Bodhi Chemical Co., Ltd. (Tianjin, China). Trifluoromethylacrylic acid (TFEMAA, analytical grade), 4-hydroxypyridine (4-HP, analytical grade), and ethyl methacrylate (EMA, analytical grade) were supplied by the Tianjin Guangfu Fine Chemical Research Institute (Tianjin, China). Ethylene glycol dimethacrylate (EGDMA, purity 99%) was acquired from Shanghai Aladdin Biochemical Technology Co., Ltd. (Shanghai, China). Acetonitrile, methanol, and acetic acid (all analytical grade) were procured from Tianjin Bodhi Chemical Co., Ltd. (Tianjin, China). All reagents were used as received except for AIBN, which was purified before use.

The following instruments were used: a 20PR-52D high-speed refrigerated centrifuge (Hitachi, Tokyo, Japan); a ZD-85 constant temperature shaker (Changzhou Guohua Electric Co., Ltd., Changzhou, Jiangsu, China); a KQ-250B ultrasonic cleaner (Kunshan Ultrasonic Instrument Co., Ltd., Kunshan, Jiangsu, China); a DF-101S magnetic stirrer (Gongyi Yuhua Instrument Co., Ltd., Gongyi, Henan, China); a DZF-series vacuum drying oven (Shanghai Yuejin Medical Instrument Co., Ltd., Shanghai, China); an AL204-IC electronic balance (Mettler Toledo, Greifensee, Switzerland); a Nicolet 6700 Fourier transform infrared (FT-IR) spectrometer (Thermo Fisher Scientific, Waltham, MA, USA); a JASCO V-630 UV-Vis spectrophotometer (Jasco, Tokyo, Japan); a Thermo ULTIMATE 3000 ultra-high-performance liquid chromatography (UHPLC) system (Thermo Fisher Scientific, Waltham, MA, USA); and a Quanta 250 environmental scanning electron microscope (FEI, Eindhoven, The Netherlands).

### 2.2. Quantum Chemical Calculation Methodology

#### 2.2.1. Molecular Model Construction and Geometry Optimization

Initial structural models for the template molecules (ANL, SNM, and SDZ) and the six functional monomers (MAA, TFEMAA, EMA, MMA, NVP, and 4-HP) were built using Gaussian View 6.0 software. The geometries of all molecules were optimized using density functional theory (DFT) at the B3LYP/6-31G+(d,p) level with the PCM solvation model (water) using the keyword scrf = (solvent = water, pcm) [19,20] in Gaussian 16 software. Frequency calculations were performed on the optimized structures to confirm that they were the true minima (no imaginary frequencies) on the potential energy surface. The imprinted binding sites of template molecules and functional monomers were analyzed based on natural bond orbital (NBO) charges and molecular electrostatic potential (MEP) analysis.

#### 2.2.2. Interaction Energy Calculation

The interaction energies (Δ*E*) for the complexes formed between template molecules and functional monomers were calculated at the B3LYP/6-31++G(d,p) level. The basis set superposition error (BSSE) was corrected using the Counterpoise (CP) method [21]. The interaction energy was calculated using Equation (1):

(1) $$\Delta E=E_{\mathrm{complex}}-E_{\mathrm{template}}-\sum E_{\mathrm{monomer}}+E_{\mathrm{BSSE}}$$

where *E*_complex_, *E*_template_, and *E*_monomer_ represent the energies of the optimized complex, the template molecule (ANL, SNM, or SDZ, depending on the system), and the functional monomer, respectively, and *E*_BSSE_ is error correction.

### 2.3. Preparation of MIPs

**Figure 2:**
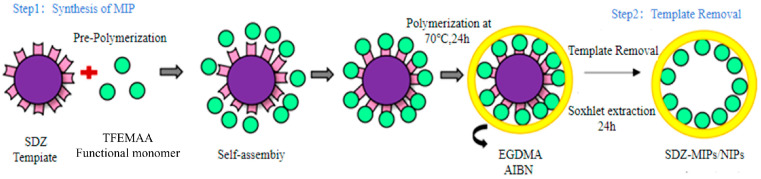
Schematic diagram of the SDZ-MIP preparation process.

SDZ molecularly imprinted polymers (MIPs) were synthesized via precipitation polymerization [22,23], and the preparation process is shown in Figure 2. Specifically, the template molecule SDZ (0.5 mmol) and the functional monomer TFEMAA (2.0 mmol) were weighed into a 100 mL brown glass bottle. Acetonitrile (50 mL) was added, and the mixture was sonicated for 30 min to ensure complete dissolution. Pre-polymerization was conducted at 4 °C for 12 h to facilitate hydrogen bond pre-assembly. Subsequently, the cross-linker EGDMA (1.92 mL) and the initiator AIBN (0.023 g) were added sequentially with thorough mixing. The reaction mixture was purged with high-purity nitrogen for 10 min to remove oxygen, sealed, and then placed in a 70 °C water bath. Polymerization proceeded for 24 h under magnetic stirring at 200 rpm. After polymerization, the resulting milky-white precipitate was centrifuged at 8000 rpm for 10 min, and the supernatant was discarded. The precipitate was dried to constant weight in an oven. The dried polymer was then transferred to a Soxhlet extractor and exhaustively washed with a methanol–acetic acid mixture (9:1, *v*/*v*) until no SDZ template was detected in the eluent. This was followed by washing with methanol to remove residual acetic acid. Finally, the completely eluted polymer was dried again to constant weight, yielding the target product: sulfadiazine molecularly imprinted polymers (SDZ-MIPs). As a control, non-imprinted polymers (NIPs) were prepared following the same procedure but in the absence of the SDZ template molecule.

### 2.4. Adsorption Performance Evaluation

#### 2.4.1. Kinetic Adsorption Experiments

SDZ-MIPs or NIPs (20 mg) were accurately weighed and added to 20 mL of a 0.5 mg/mL SDZ solution (in triplicate). At predetermined time intervals (5, 10, 20, 30, 60, and 120 min), 1 mL of the supernatant was withdrawn, filtered through a 0.22 μm membrane, and diluted appropriately, and its absorbance was measured at 268 nm (λmax for SDZ) using a UV-Vis spectrophotometer [24]. The adsorption capacity (*Q_t_*, mg/g) at time t was calculated using Equation (2):

(2) $$Q_{t}=\frac{(C_{0}-C_{t})V}{m}\times 1000$$

where *C*_0_ and *C_t_* (mg/mL) are the initial concentration and the concentration at time *t*, respectively; *V* (mL) is the solution volume; and *m* (g) is the mass of the polymer. The kinetic adsorption curve for SDZ-MIPs was plotted.

#### 2.4.2. Isothermal Adsorption Experiments

SDZ-MIPs or NIPs (2 mg) were accurately weighed and added to 2 mL of SDZ solutions with varying initial concentrations (0.05, 0.1, 0.3, 0.5, 0.8, and 1.0 mg/mL) in triplicate. After adsorption for 2 h, the mixtures were centrifuged. The supernatant was diluted and analyzed by UV-Vis spectroscopy to determine the equilibrium concentration. The equilibrium adsorption capacity (*Q_e_*) was calculated using a form of Equation (2), and the isothermal adsorption curve for SDZ-MIPs was constructed.

#### 2.4.3. Selectivity Experiments

To evaluate selectivity, compounds structurally similar to SDZ (SNM and SMX) and a structurally dissimilar compound (SMM) were selected as interferents. SDZ-MIPs or NIPs (2 mg) were added to 2 mL of a 0.5 mg/mL solution of each target compound (SDZ, SNM, SMX, and SMM) in triplicate. After 2 h of adsorption, the mixtures were processed and analyzed as described in Section 2.4.1 to calculate the adsorption capacity (*Q_e_*) for each compound. The equation for the distribution coefficients of SDZ, SNM, SMX and SMM is

(3) $$K_{d}=\frac{Q_{e}}{C_{e}}$$

where *K_d_* (mL/g) is the distribution coefficient, *Q_e_* (mg/g) is the adsorption capacity of SDZ, and *C_e_* (mg/mL) is the residual concentration of SDZ.

The selectivity coefficients of MIPs and NIPs for SDZ compared with SNM, SMX or SMM were calculated using

(4) $$K=\frac{K_{d}(\mathrm{SDZ})}{K_{d}(\mathrm{SNM}/\mathrm{SMX}/\mathrm{SMM})}$$

where *K* is the selectivity coefficient.

The relative selectivity coefficient was calculated using

(5) $$K^{\prime }=\frac{K_{\mathrm{MIP}}}{K_{\mathrm{NIP}}}$$

where *K*′ is the relative selectivity coefficient, while *K*_MIP_ and *K*_NIP_ are the selectivity coefficients of MIPs and NIPs, respectively.

#### 2.4.4. Comparison with Other Adsorbents

The widely used HLB filler (a copolymer of divinylbenzene and N-vinylpyrrolidone) employed in standard methods was selected for comparison. SDZ-MIPs and the HLB filler (2 mg each) were separately added to 2 mL of a 0.5 mg/mL SDZ aqueous solution (in triplicate). After 2 h of adsorption, the mixtures were processed, and the adsorption capacity was determined as per Section 2.4.1.

#### 2.4.5. Application in Real Water Samples

Real water samples, including water from the Hunhe River and the Puhe River (both in Shenyang, China), as well as tap water, were tested. Initial analysis confirmed the absence of detectable SDZ in these samples. Subsequently, SDZ-MIPs (2 mg) were added to 2 mL aliquots of each water sample spiked with SDZ at a concentration of 0.5 mg/mL. After 2 h of adsorption, the mixtures were centrifuged, and the supernatant was filtered through a 0.22 μm membrane before analysis to determine the adsorption capacity.

## 3. Results and Discussion

### 3.1. DFT-Based Analysis of the Molecular Imprinting Pre-Assembly System

To systematically investigate structure-dependent recognition behavior, three template molecules with increasing structural complexity were analyzed: ANL as a simplified aromatic amine model, SNM as the sulfonamide core scaffold, and SDZ as the target molecule. This hierarchical design was used to elucidate template–monomer matching rules in sulfonamide imprinting systems and to provide mechanistic support for the final selection of the SDZ imprinting system.

#### 3.1.1. Optimization of Geometric Configurations and Charge Distribution Analysis

**Figure 3:**
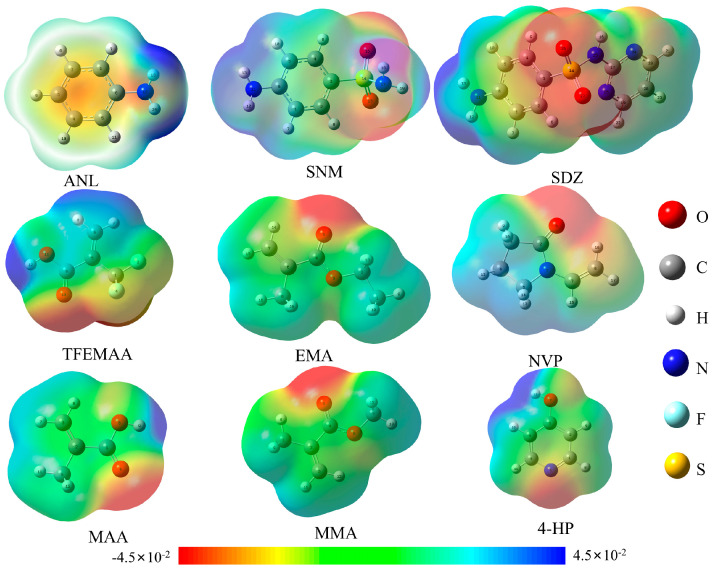
MEP distributions of the template molecules and functional monomers.

The optimized geometric configurations and the MEP for the template molecules (ANL, SNM, and SDZ) and the six functional monomers (MAA, TFEMAA, EMA, MMA, NVP, and 4-HP) are presented in Figure 3.

As indicated by the color scale in Figure 3, regions in red represent negative electrostatic potential (electrophilic sites), while blue regions indicate positive potential (nucleophilic sites) [25,26]. The MEP distributions, together with NBO charge analysis, were used to identify plausible hydrogen bond donor/acceptor sites and to guide the construction of candidate template–monomer complexes for subsequent DFT optimization. According to fundamental principles, the interaction between strongly nucleophilic and electrophilic sites typically leads to the formation of more active non-covalent bonds [27]. Therefore, the theoretical binding sites based on NBO charge analysis for ANL, SNM, and SDZ with the various monomers are summarized in Table 1.

#### 3.1.2. Interaction Energy and Hydrogen Bond Analysis of Template–Monomer Complexes

**Figure 4:**
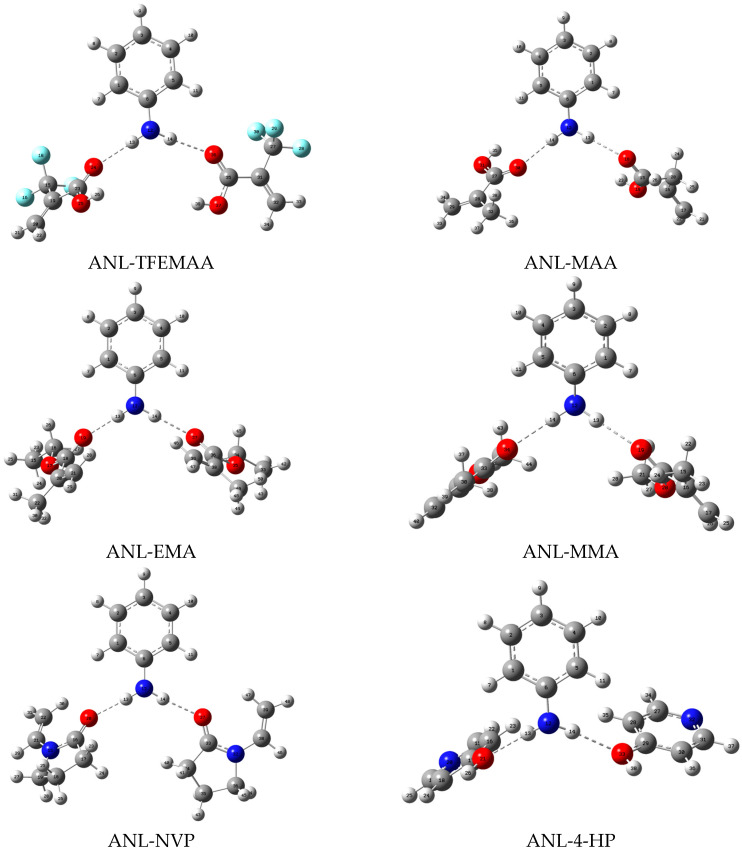
Geometric configurations of the stable complexes formed between ANL and the functional monomers.

**Figure 5:**
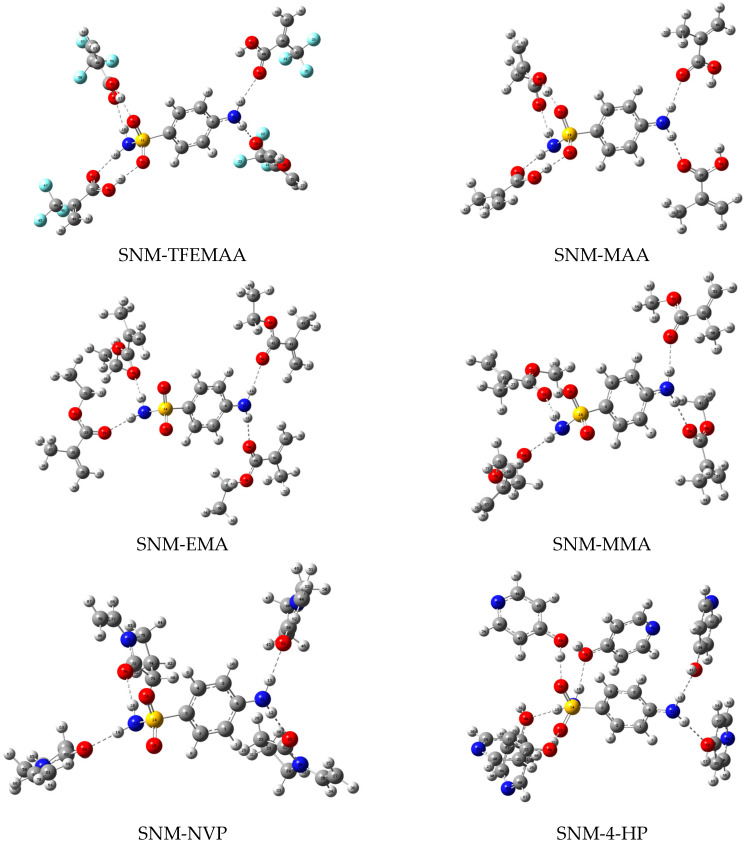
Geometric configurations of the stable complexes formed between SNM and the functional monomers.

**Figure 6:**
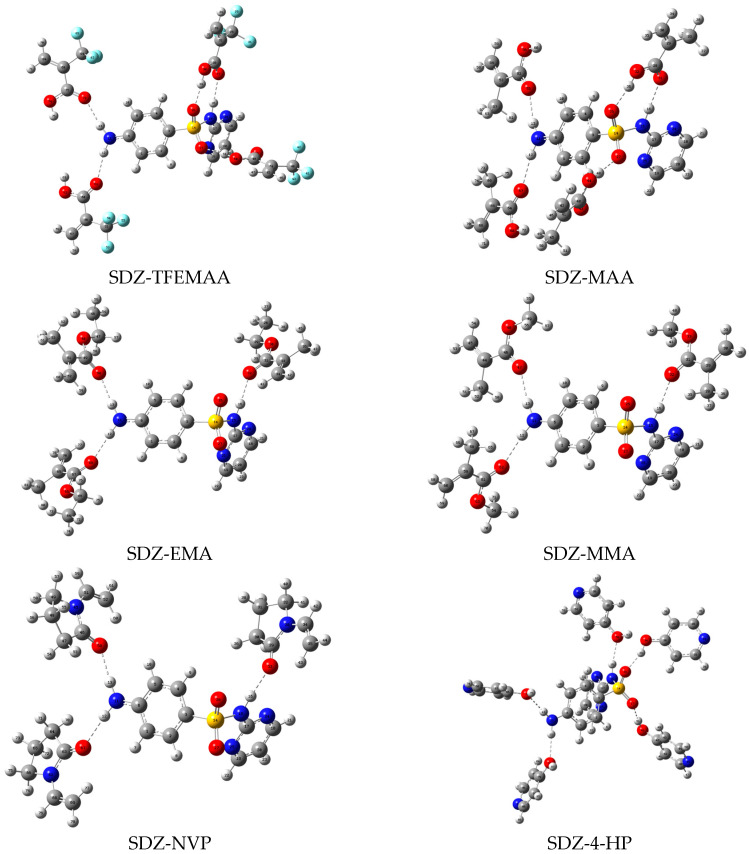
Geometric configurations of the stable complexes formed between SDZ and the functional monomers.

Based on the proton donor/acceptor distribution analyzed in Section 3.1.1, stable configurations of complexes between the template molecules and functional monomers at various molar ratios were simulated using Gaussian 16 at the B3LYP/6-31G+(d,p) level. After screening and multiple rounds of optimization, the stable conformations of the complexes were finally obtained, as shown in Figure 4, Figure 5 and Figure 6. Structural parameters such as the number, length, and angle of hydrogen bonds within each complex were extracted via single-point energy calculations. These parameters, integrated with the computed interaction energies (ΔE), are systematically presented in Table 2 to elucidate the binding characteristics.

In the ANL system, the functional monomers were ranked by the absolute value of their interaction energy (|ΔE|, where a larger negative value indicates stronger, more favorable interaction) as follows: MMA > NVP > EMA > MAA > TFEMAA > 4-HP. According to intermolecular interaction theory, a larger absolute value of interaction energy (all interactions here are exothermic) indicates stronger non-covalent interactions (e.g., hydrogen bonding and electrostatic forces) between the template and the functional monomer, resulting in a more thermodynamically stable complex [22]. Acrylate monomers (MMA and EMA) demonstrated superior performance compared to acrylic acid monomers (MAA and TFEMAA). Among the N-heterocyclic monomers, NVP performed well, while 4-HP was the least effective. This trend can be attributed to the relatively low positive charge on the amino N-H of ANL and the absence of strong acceptor sites, thus requiring pairing with monomers possessing a weak acceptor character to avoid competitive binding. Carboxylic acid monomers (MAA and TFEMAA), featuring both a hydrogen bond donor (-OH) and acceptor (C=O) within their -COOH group, present a complication: the -OH group can compete with ANL’s N-H bonds for binding sites. As seen in Table 2, although the C=O group can interact with ANL’s N-H bonds, the resulting bond lengths are relatively long (2.12–2.16 A). In contrast, acrylate monomers only possess a hydrogen bond acceptor (C=O, from the ester group) without an interfering donor, and the C=O group carries a higher negative charge, facilitating the formation of shorter hydrogen bonds with ANL’s N-H bonds. The C=O group in NVP’s pyrrolidone ring, with oxygen charges from −0.649 to −0.689, formed the shortest hydrogen bonds (2.053–2.059 A), likely due to the flexibility of its five-membered ring that allows optimal angular alignment with ANL’s planar structure. The weakest binding with 4-HP resulted from its -OH group tending to form an O-H∙∙∙N hydrogen bond with ANL’s amine nitrogen. However, the weak basicity of ANL’s nitrogen led to significant bond angle distortion (~174°), reducing interaction stability.

The SNM system revealed a markedly different trend: |ΔE| decreased in the order of TFEMAA > MAA > 4-HP > MMA > NVP > EMA. Here, carboxylic acid monomers outperformed acrylate monomers, and 4-HP became the best among N-heterocyclics, while NVP’s performance declined. SNM contains a sulfonamide group (-SO_2_-NH-) in addition to the aniline moiety, providing two strong acceptors (S=O) and two strong donors (N-H), along with the two N-H donors from the aniline amino group, totaling six potential binding sites. The tetrahedral geometry of the sulfonamide allows its S=O bonds to simultaneously engage with two -OH groups, forming dual hydrogen bonds. Carboxylic acid monomers can leverage this by forming dual hydrogen bonds between their -OH and -C=O groups and the S=O and N-H sites of the sulfonamide, creating a highly stable complex. The calculated S=O∙∙∙H-O hydrogen bond lengths averaged 1.75 Å. Acrylate monomers suffer a dual disadvantage: ester oxygen possesses lower electron density, weakening the interaction with SNM’s strong S=O acceptors, and they can only form hydrogen bonds with the four N-H donors, resulting in less stable complexes. For N-heterocycles, 4-HP excelled because the benzene ring in SNM, which is attached to the strongly electron-withdrawing sulfonyl group, has reduced electron density. The pyridine ring in 4-HP is an electron-deficient π-system, enabling strong π-π stacking interactions. Furthermore, pyridinic nitrogen acts as an acceptor for N-H∙∙∙N bonds, and its -OH group serves as both a strong donor and acceptor, allowing flexible engagement with multiple sites on SNM (O-H∙∙∙O=S or N-H∙∙∙O). NVP performed slightly worse because; although its strong carbonyl acceptor can form N-H∙∙∙O=C bonds, the bulky pyrrolidone ring introduces significant steric hindrance, limiting effective integration into SNM’s dense network of binding sites and compromising both the quantity and quality of hydrogen bonds.

In the SDZ system, the ranking of |ΔE| was TFEMAA > MAA > 4-HP > MMA > NVP > EMA, mirroring the SNM system with high consistency. Structurally, SDZ can be viewed as an SNM core with a pyrimidine ring attached to the sulfonamide nitrogen. Both share the critical -SO_2_-NH- group and a para-amino moiety. Although SDZ possesses an additional pyrimidine ring, the simulation results indicate that under the current screening conditions, the formation of the imprinting site is predominantly governed by interactions with the common -SO_2_-NH- core. The unique pyrimidine ring of SDZ did not appear to significantly alter the functional monomer selection hierarchy. This key finding suggests that for the rational design of MIPs targeting sulfonamide drugs, priority should be given to functional monomers capable of forming multiple strong hydrogen bonds with both the -SO_2_-NH- and -NH_2_ groups to achieve highly stable pre-polymerization complexes, with the common sulfonamide scaffold being the primary driver of selectivity.

The significance of the interaction energy data presented in Table 2 lies not merely in the absolute values for individual complexes but in the systematic and comparative analysis across three template molecules (ANL, SNM, and SDZ) and six functional monomers from three distinct categories (carboxylic acids, acrylates, and N-heterocycles). This comprehensive dataset allows, for the first time, a deconvolution of the contributions of different structural fragments—such as the amino group, the sulfonamide core, and the pyrimidine ring—to the overall template–monomer interaction. The distinct ranking of monomer affinities observed for ANL (acrylate > acid), SNM (acid > acrylate), and the consistent top performer (TFEMAA) for both SNM and SDZ provides mechanistic insight into how the sulfonamide backbone dictates monomer selection. Such a systematic evaluation goes beyond previous studies that typically only report the optimal combination for a single target and establishes a rational framework for designing imprinted materials tailored to sulfonamide antibiotics.

**Figure 7:**
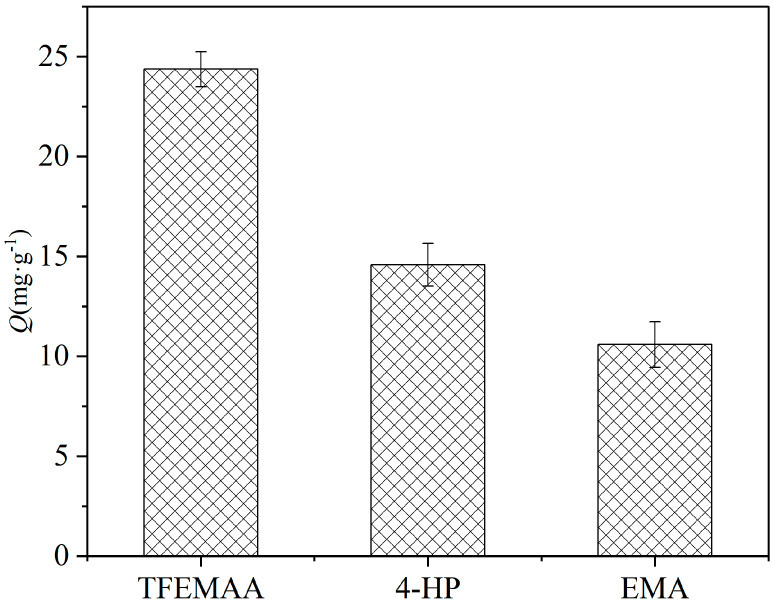
Comparison of adsorption capacities of molecularly imprinted polymers prepared with different functional monomers.

To experimentally validate the reliability of the computational screening, we selected the monomer with the highest interaction energy (TFEMAA), the monomer with medium interaction energy (4-HP), and the monomer with the lowest interaction energy (EMA) to synthesize the corresponding molecularly imprinted polymers under identical conditions. Their adsorption capacities toward SDZ were compared at an initial concentration of 0.5 mg/mL with an adsorption time of 2 h. As shown in Figure 7, the adsorption capacities followed the order of TFEMAA (24.12 mg/g) > 4-HP (18.67 mg/g) > EMA (15.88 mg/g), which is fully consistent with the theoretical interaction energy ranking (TFEMAA > 4-HP > EMA). This result strongly confirms that the DFT-calculated interaction energy serves as a reliable indicator for functional monomer screening. It also demonstrates that 4-HP, which has medium interaction energy, can still form effective recognition sites in the actual imprinting process, though its performance is inferior to that of the optimal monomer TFEMAA.

### 3.2. Characterization of the Polymers

#### 3.2.1. Morphological Characterization

**Figure 8:**
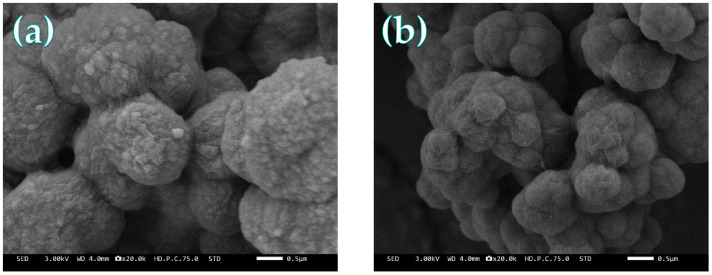
SEM images of (**a**) MIPs and (**b**) NIPs.

The microstructures of the MIPs and NIPs were examined using scanning electron microscopy (SEM). As shown in Figure 8, both polymers exhibited well-defined, uniform nanoscale spherical morphologies with high sphericity. The surface of the MIPs’ microspheres was notably rough and populated with abundant pores and cavities, which are the imprinting sites left after template removal (Figure 8a). This microporous architecture is crucial, providing accessibility and favorable pathways for SDZ adsorption. In contrast, the NIP surface appeared relatively smoother (Figure 8b), confirming the structural distinction imparted by the imprinting process.

#### 3.2.2. FT-IR Spectroscopic Characterization

**Figure 9:**
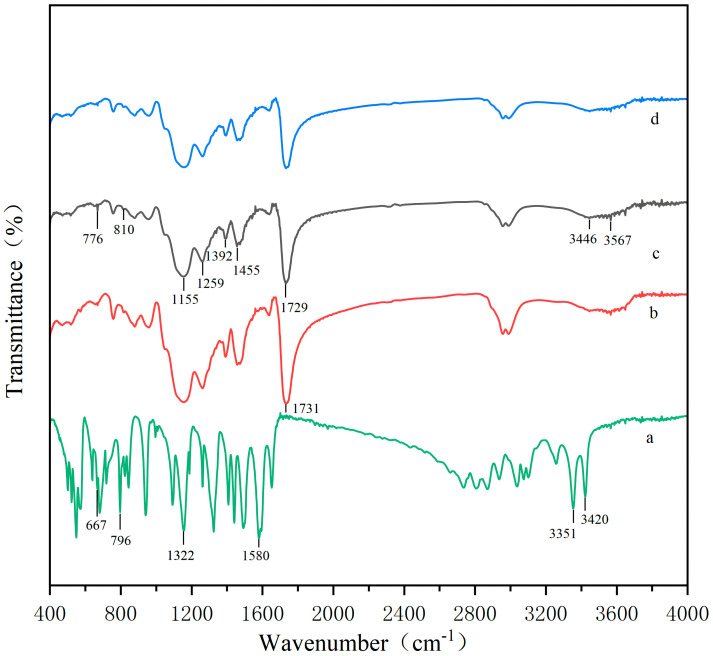
FT-IR spectra of (**a**) SDZ, (**b**) MIPs before elution, (**c**) NIPs, and (**d**) MIPs after elution.

Infrared spectroscopy is an effective method to study organic molecules with asymmetric structures and polar functional groups. The structural changes in polymers induced by template incorporation and removal can be investigated by infrared spectroscopy. As shown in Figure 9, there are some identical absorption peaks in the spectra of MIPs before elution (Figure 9b), MIPs after elution (Figure 9d), and NIPs (Figure 9c). Among them, the stretching vibration peak of O–H appears around 3446 cm^−1^, while the stretching vibration peak of C=O from the functional monomer and cross-linker is observed at approximately 1731 cm^−1^, and the stretching vibration absorption peaks of C–O–C in EGDMA appears at 1259 cm^−1^ and 1151 cm^−1^. These common features confirm the successful copolymerization of the functional monomer and cross-linker in all polymer matrices [28].

According to the spectrum of SDZ (Figure 9a), characteristic absorption peaks are observed at 3351 cm^−1^ and 3420 cm^−1^ (stretching vibrations of –NH_2_), 1580 cm^−1^ and 1322 cm^−1^ (C=N and C–N vibrations), and 796 cm^−1^ and 667 cm^−1^ (O=S=O stretching). In the spectrum of pre-eluted MIPs (curve b), additional peaks appear at 667 cm^−1^ and 810 cm^−1^, which correspond to the characteristic absorption of the –SO_2_–NH– group of the embedded SDZ template. The presence of these SDZ-specific peaks in curve b indicates that the template molecule was successfully incorporated into the polymer matrix during polymerization.

After elution (Figure 9d), these characteristic absorption peaks at 667 cm^−1^ and 810 cm^−1^ completely disappear, and the spectrum becomes nearly identical to that of NIPs (Figure 9c). At the same time, the C=O stretching vibration peak shifts slightly from its position in pre-eluted MIPs to 1729 cm^−1^, which is similar to that in NIPs. The complete disappearance of the SDZ characteristic peaks provides direct spectroscopic evidence that the template molecule imprinted in the polymer can be successfully removed by washing with acetic acid and methanol. The results demonstrate that SDZ was imprinted into the polymer matrix and subsequently eluted, leaving behind specific recognition cavities complementary to the SDZ molecular structure.

### 3.3. Adsorption Performance of the Polymers

#### 3.3.1. Dynamic Adsorption and Kinetic Analysis

**Figure 10:**
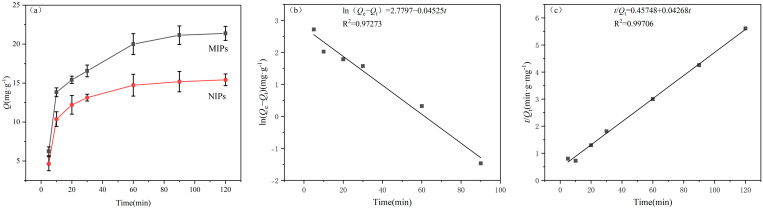
(**a**) Adsorption kinetics of MIPs and NIPs. Fitting curves for (**b**) pseudo-first-order and (**c**) pseudo-second-order kinetic models.

The adsorption process of SDZ onto the MIPs was evaluated using pseudo-first-order and pseudo-second-order kinetic models. The adsorption kinetics of SDZ onto MIPs and NIPs were investigated at a fixed SDZ concentration (20 mg/L). As shown in Figure 10a, adsorption increased rapidly within the first 90 min before gradually plateauing at equilibrium. Both MIPs and NIPs reached saturation at approximately the same time. This two-phase adsorption likely corresponds to initial fast occupation of readily accessible (“shallow”) sites, followed by slower diffusion and binding to less accessible (“deep”) imprinting cavities within the MIPs.

To elucidate the adsorption mechanism, the kinetic data were fitted to pseudo-first-order and pseudo-second-order models (Equations (6) and (7)).

(6) $$\ln\left(Q_{e}-Q_{t}\right)=\ln Q_{e}-k_{1}t$$

(7) $$t/Q_{t}={\left(k_{2}{Q_{e}}^{2}\right)}^{-1}+t/Q_{e}$$

where *Q_e_* is the equilibrium adsorption capacity (mg·g^−1^); *Q_t_* is the adsorption capacity at time *t* (mg·g^−1^); *t* is the time (min); *k*_1_ is the rate constant of the pseudo-first-order kinetic model (min^−1^); and *k*_2_ is the rate constant of the pseudo-second-order kinetic model (g·mg^−1^·min^−1^).

The fitting results (Figure 10b,c) revealed a significantly higher correlation coefficient for the pseudo-second-order model (R^2^ = 0.99706) compared to the pseudo-first-order model (R^2^ = 0.97273). The superior fit of the pseudo-second-order model strongly indicates that chemisorption is the rate-limiting step [29], consistent with the multiple hydrogen bond interactions predicted by our DFT calculations between TFEMAA and SDZ. The theoretical equilibrium adsorption capacity (*Q_e_*, cal = 23.43 mg·g^−1^) derived from this model closely matched the experimental value (21.38 mg·g^−1^).

#### 3.3.2. Isothermal Adsorption and Model Analysis

**Figure 11:**
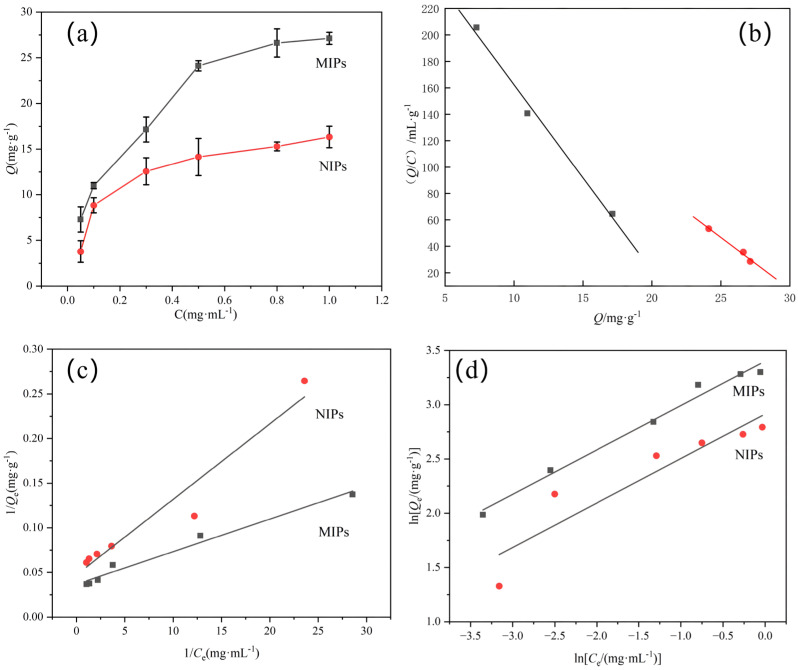
(**a**) Static adsorption isotherms of MIPs and NIPs for SDZ. (**b**) Scatchard plot analysis for SDZ adsorption onto MIPs. Fitting curves of the (**c**) Langmuir model and the (**d**) Freundlich model.

Static adsorption isotherms (Figure 11a) demonstrated that the adsorption capacity of both MIPs and NIPs increased with the SDZ concentration. However, the MIPs consistently exhibited higher adsorption capacity across all concentrations, which is directly attributed to the presence of specific imprinted cavities.

The heterogeneity of binding sites within the MIPs was quantitatively assessed using the Scatchard model (Equation (8)).

(8) $$\frac{Q}{C}=\frac{Q_{\max}-Q}{K}$$

where *Q* is the adsorption capacity of the MIPs for SDZ (µg/mg); *C* is the equilibrium concentration of SDZ in the adsorption solution (µg/mL); *Q*_max_ is the maximum apparent binding capacity of the binding sites (µg/mg); and *K* is the equilibrium dissociation constant of the binding sites (µg/mL).

The results of the Scatchard analysis are shown in Figure 11b. The Scatchard plot for MIPs and NIPs shows two straight lines with different slopes [30]. The calculated dissociation constants (*K*_d_) and maximum binding capacities (*Q*_max_) were 0.065 mg/mL and 20.10 mg/g for the high-affinity sites and 20.72 mg/mL and 60.5 mg/g for the low-affinity sites, respectively. This heterogeneous site distribution is a hallmark of molecularly imprinted materials and arises from thermodynamic and kinetic variations during the polymerization process. The high-affinity sites (low *K*_d_) correspond to well-defined cavities formed by optimal template–monomer complexes, aligning with the strong hydrogen bond networks identified in simulations. The more abundant low-affinity sites (high *K*_d_) likely represent non-specific or partially complementary cavities resulting from the stochastic nature of free-radical polymerization.

The adsorption processes of MIPs and NIPs were further analyzed by fitting the experimental data with both the Langmuir and Freundlich adsorption models. The formulas for the two adsorption models are shown in Equations (9) and (10), respectively.

(9) $$\frac{1}{Q_{e}}=\frac{1}{K_{1}Q_{m}}\times \frac{1}{C_{e}}+\frac{1}{Q_{m}}$$

(10) $$\ln Q_{e}=\ln K_{2}+\frac{1}{n}\ln C_{e}$$

where *C_e_* is the equilibrium concentration (mg/mL), *Q_e_* is the equilibrium adsorption capacity (mg/g), *_Qm_* is the maximum adsorption capacity (mg/g), and *K*_1_, *K*_2_, and *n* are constants.

The adsorption isotherm is assessed by the Langmuir and Freundlich isothermal models (Figure 11c,d), and the involved fitting parameters are listed in Table 3. For MIPs, the Freundlich model (R^2^ = 0.9854) provided a slightly better fit compared to the Langmuir model (R^2^ = 0.9783), confirming that the active site distribution on MIPs is heterogeneous, which is consistent with the Scatchard analysis revealing multiple types of binding sites. The Freundlich exponent *n* = 2.44 (1/*n* = 0.409) indicates favorable adsorption. For NIPs, the Langmuir model (R^2^ = 0.9413) showed a better fit than the Freundlich model (R^2^ = 0.8644), suggesting relatively homogeneous binding sites on the non-imprinted polymer surface, which aligns with the single linear Scatchard plot observed for NIPs.

To further bridge the computational and experimental results, the adsorption free energy (Δ*G*°) was calculated from the Langmuir isotherm constant using Equation (11).

(11) $$\Delta G^{\circ }=-RT\ln K$$

where *R* is the universal gas constant (8.314 J mol^−1^ K^−1^) and *T* is the temperature is Kelvin.

The calculated Δ*G*° values were –2.30 kJ·mol^−1^ for MIPs and –1.85 kJ·mol^−1^ for NIPs. The negative values confirm the spontaneous nature of the adsorption process, and the more negative Δ*G*° for MIPs indicates stronger affinity toward SDZ, consistent with the higher adsorption capacity observed.

#### 3.3.3. Selective Adsorption Experiments 

**Figure 12:**
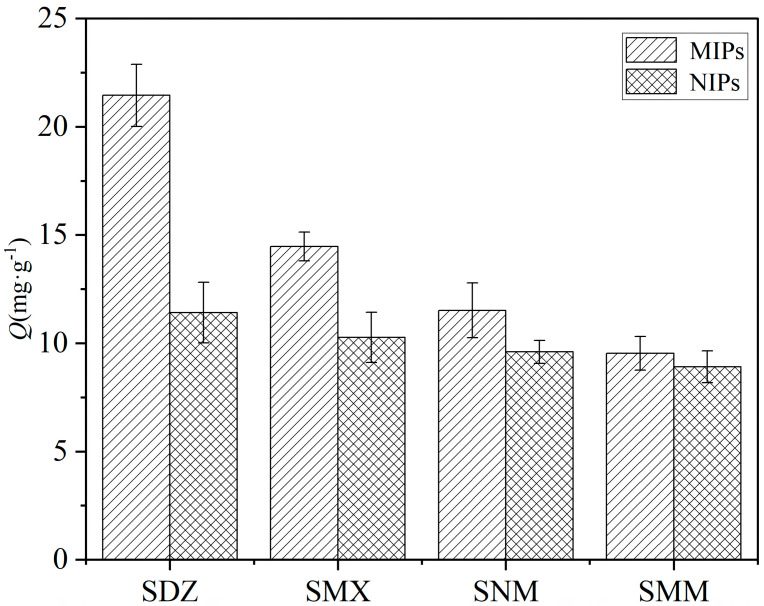
Selective adsorption capacities of MIPs and NIPs for SDZ and its analogs.

Selectivity was evaluated against structural analogs (SNM and SMX) and a non-analog (SMM). The adsorption capacities of MIPs and NIPs for SDZ and its analogs were determined under identical conditions. As shown in Figure 12, the MIPs showed the highest adsorption for the template SDZ, followed by its closer analog SNM, then SMX, and finally SMM. This hierarchy reflects the degree of structural complementarity to the imprinted cavity. While NIPs also adsorbed these compounds due to non-specific interactions, the significantly higher adsorption of SDZ by MIPs unequivocally demonstrates imprinting-induced specificity.

To quantitatively assess selectivity, the distribution coefficient (K_d_), selectivity coefficient (k), and relative selectivity coefficient (k′) were calculated, and the results are summarized in Table 4. The *K_d_* value of MIPs for SDZ (46.92 mL/g) was markedly higher than those for SNM (30.71 mL/g), SMX (24.16 mL/g), and SMM (19.82 mL/g). The selectivity coefficients (k) of MIPs, defined as the ratio of K_d_(SDZ) to *K_d_*(analog), were 1.53 for SNM, 1.94 for SMX, and 2.37 for SMM. Notably, the k value increased as the structural difference between the analog and SDZ became more pronounced, which is consistent with the molecular recognition mechanism of imprinted cavities. For NIPs, the *K_d_* values of the four compounds were much closer (18.49–23.94 mL/g), and the corresponding k values were near unity (1.12–1.29), confirming that NIPs lack selective recognition ability.

The relative selectivity coefficient (*K*′), defined as the ratio of *K*_MIP_ to *K*_NIP_, quantifies the imprinting effect on selectivity enhancement. The k′ values for SNM, SMX, and SMM were 1.71, 2.00, and 2.27, respectively, and were all greater than one. This confirms that the superior selectivity of MIPs originates from the specific imprinted cavities complementary to SDZ’s molecular structure, which were formed during the polymerization process. NIPs, lacking such spatial structures, exhibit only non-specific adsorption on irregular binding sites.

#### 3.3.4. Comparison with Commercial Adsorbents

The performance of SDZ-MIPs was benchmarked against a widely used commercial adsorbent, the HLB filler. SDZ-MIPs exhibited a markedly higher adsorption capacity (25.25 mg/g) compared to HLB (15.50 mg/g). This superior performance underscores the advantage of tailored molecular recognition over general hydrophobic interaction, highlighting the potential of MIPs for the selective extraction of SDZ from complex matrices.

#### 3.3.5. Application in Real Water Samples

The practical applicability of SDZ-MIPs was tested using spiked real water samples: Hunhe River water, Puhe River water, and tap water (Table 5). While the adsorption capacity in the more complex river water matrices (17.0 and 15.5 mg/g) was somewhat reduced compared to that in tap water (25.25 mg/g) or the pure solution, the MIPs retained significant adsorption performance. This demonstrates a robust anti-interference capability against common matrix constituents (e.g., dissolved organic matter and ions), confirming their potential for real-world environmental remediation applications.

#### 3.3.6. Potential for Membrane Separation Applications

The synthesized SDZ-MIPs, characterized by their uniform spherical morphology, high selectivity, and matrix tolerance, present a promising material foundation for advanced membrane separation. The most common approaches include: (i) mixed matrix membranes (MMMs), where MIPs particles are embedded into a porous polymer matrix (e.g., polyethersulfone and polyvinylidene fluoride) during membrane casting, creating a composite membrane that combines selective adsorption with continuous fluid flow [31,32]; (ii) surface coating or grafting, where a thin MIP layer is deposited onto a pre-formed support membrane or covalently attached to membrane pores, enabling selective recognition at the membrane/feed interface; and (iii) membrane adsorbers, where MIP-functionalized membranes are used in dead-end or cross-flow filtration modes, capturing target molecules during permeation and allowing subsequent elution for regeneration. In such configurations, the membrane operates continuously until the imprinted sites approach saturation. At that point, an online regeneration step—such as flushing with a suitable solvent (e.g., methanol/acetic acid mixture)—can restore the membrane’s binding capacity, enabling cyclic operation. This concept of regenerable MIP-based membranes has been successfully demonstrated in several studies, confirming their practical feasibility. Such MIP-functionalized membranes are anticipated to achieve high-selectivity permeation or rejection of target antibiotics like SDZ while maintaining adequate flux, offering a strategic solution for the targeted removal and concentration of sulfonamide antibiotics from complex wastewater streams (e.g., pharmaceutical or aquaculture effluent). Future work will focus on optimizing MIP incorporation methods, tailoring membrane morphology, and evaluating long-term operational stability.

## 4. Conclusions

This study demonstrated the rational design of sulfadiazine (SDZ)-imprinted polymers guided by density functional theory (DFT) calculations, which identified trifluoroethyl methacrylate (TFEMAA) as the optimal functional monomer at a template-to-monomer molar ratio of 1:4. The simulations elucidated the critical hydrogen-bonding matching mechanism, providing a reliable theoretical framework that streamlines the synthesis process. Experimentally, the synthesized SDZ-MIPs achieved adsorption equilibrium within 90 min and exhibited superior selectivity for SDZ over its structural analogs. Scatchard model analysis confirmed the presence of heterogeneous binding sites with distinct affinities within the MIPs, consistent with the predicted distribution of imprinting cavities. In practical tests using real water matrices, the SDZ-MIPs outperformed a conventional commercial HLB sorbent, demonstrating robust anti-interference capability and significant application potential for targeted SDZ removal. These findings provide not only a high-performance adsorbent for the selective remediation of SDZ-contaminated water—particularly from pharmaceutical or aquaculture sources—but also a fundamental material and theoretical foundation. This work lays the groundwork for the subsequent development of SDZ-imprinted composite membranes, paving the way for the scalable application of molecular imprinting technology in advanced, high-selectivity membrane separation systems for environmental remediation.

## Figures and Tables

**Table 1:** NBO charges of key atoms and potential hydrogen-bonding sites for template molecules and functional monomers.

Template/Monomer	Hydrogen Bond Donor	NBO Charge	Hydrogen Bond Acceptor	NBO Charge	Number of H-Bonds
ANL	H13, H14	0.400, 0.400	—	—	2
SNM	H12, H13	0.414, 0.415	O15	−0.646	6
	H18, H19	0.444, 0.447	O16	−0.618	
SDZ	H12, H13	0.416, 0.416	O26	−0.598	5
	H16	0.489	O27	−0.555	
TFEMAA	H12	0.535	O10	−0.516	2
MAA	H9	0.521	O4	−0.546	2
EMA	—	—	O5	−0.548	1
MMA	—	—	O5	−0.572	1
NVP	H12	0.511	N6	−0.292	3
			O7	−0.703	
4-HP	—	—	O6	−0.633	1

**Table 2:** Interaction energy and hydrogen-bonding parameters between template molecules and functional monomers.

Molar Ratio	Number of H-Bonds	Interaction Site	NBO Charge	Bond Length (nm)	Bond Angle (°)	Interaction Energy (KJ/mol)
ANL:MAA 1:2	2	N12-H13---O18=C15	0.463–−0.547	2.12	179.12	−6.61
		N12-H14---O30=C27	0.463–−0.538	2.13	141.45	
ANL:TFEMAA 1:2	2	N12-H13---O24=C23	0.465–−0.505	2.16	179.06	−5.33
		N12-H14---O36=C35	0.466–−0.520	2.16	175.82	
ANL:EMA 1:2	2	N12-H13---O19=C18	0.476–−0.627	2.09	178.67	−7.29
		N12-H14---O37=C36	0.466–−0.520	2.09	178.80	
ANL:MMA 1:2	2	N12-H13---O19=C18	0.472–−0.569	2.11	176.24	−20.17
		N12-H14---O30=C27	0.472–−0.557	2.11	177.08	
ANL:4-HP 1:2	2	N12-H13---O21-H26	0.456–−0.720	2.17	173.85	−2.94
		N12-H14---O33-H38	0.450–−0.716	2.17	174.01	
ANL:NVP 1:2	2	N12-H13---O20=C16	0.483–−0.649	2.05	176.46	−10.87
		N12-H14---O37=C33	0.480–−0.689	2.06	175.28	
SNM:MAA 1:4	6	N11-H12---O23=C20	0.502–−0.560	2.04	171.51	−57.16
		N12-H13---O47=C44	0.503–−0.557	2.04	171.79	
		S14=015---H40-036	−0.649–0.597	1.76	173.94	
		S14=016---H64-060	−0.632–0.614	1.77	172.80	
		N17-H18---O59=C56	0.508–−0.575	1.95	168.96	
		N17-H19---O35=C32	0.519–−0.573	1.99	172.81	
SNM:TFEMAA 1:4	6	N11-H12---O29=C28	0.492–−0.488	2.07	176.09	−57.83
		N12-H13---O41=C40	0.488–−0.520	2.09	165.13	
		S14=015---H55-054	−0.650–0.607	1.73	174.40	
		S14=016---H67-066	−0.623–0.623	1.74	173.58	
		N17-H18---O65=C64	0.510–−0.546	2.00	166.91	
		N17-H19---O53=C52	0.524–−0.550	1.99	170.91	
SNM:EMA 1:4	4	N11-H12---O60=C59	0.506–−0.664	2.06	168.21	−24.74
		N12-H13---O42=C41	0.506–−0.609	2.05	167.95	
		N17-H18---O78=C77	0.525–−0.665	1.98	171.46	
		N17-H19---O69=C68	0.523–−0.614	1.99	170.39	
SNM:MMA 1:4	4	N11-H12---O39=C38	0.494–−0.597	2.05	171.67	−49.63
		N12-H13---O41=C40	0.501–−0.568	2.04	174.10	
		N17-H18---O24=C23	0.525–−0.611	1.99	169.99	
		N17-H19---O69=C68	0.527–−0.585	1.99	178.79	
SNM:4-HP 1:6	6	N11-H12---O62-H67	0.522–−0.729	2.10284	177.45	−55.18
		N11-H13---O38-H43	0.526–−0.726	2.09781	178.40	
		S14=015---H31-026	−0.685–0.615	1.78837	175.29	
		S14=016---H55-050	−0.634–0.623	1.75640	171.23	
		N17-H18---O74-H79	0.522–−0.729	2.04403	171.27	
		N17-H19---O86-H91	0.526–−0.726	2.01842	176.83	
SNM:NVP 1:4	4	N11-H12---O25=C21	0.506–−0.620	1.99	175.61	−35.28
		N12-H13---O42=C38	0.505–−0.670	1.99	174.76	
		N17-H18---O76=C72	0.522–−0.611	1.94	177.75	
		N17-H19---O59=C55	0.522–−0.694	1.93	178.30	
SDZ:MAA 1:4	5	N11-H12---O67=C64	0.509–−0.608	2.06	165.12	−47.56
		N12-H13---O55=C52	0.509–−0.611	2.06	165.30	
		S14=O26---H36-O32	−0.657–0.609	1.77	174.41	
		S14=O27---H48-O44	−0.633–0.613	1.80	170.28	
		N15-H16---O31=C28	0.563–−0.549	1.89	175.94	
SDZ:TFEMAA 1:4	5	N11-H12---O61=C60	0.499–−0.533	2.07	169.55	−49.75
		N12-H13---O73=C72	0.499–−0.533	2.07	170.82	
		S14=O26---H39-O38	−0.656–0.614	1.75	176.08	
		S14=O27---H51-O50	−0.602–0.629	1.77	167.50	
		N15-H16---O37=C36	0.563–−0.509	1.92	177.12	
SDZ:EMA 1:3	3	N11-H12---O68=C67	0.507–−0.640	2.02	175.57	−19.56
		N12-H13---O50=C49	0.503–−0.635	2.02	174.48	
		N15-H16---O32=C31	0.552–−0.599	1.94	174.96	
SDZ:MMA 1:3	3	N11-H12---O62=C61	0.500–−0.558	2.03	171.84	−38.12
		N12-H13---O47=C46	0.507–−0.627	2.03	170.70	
		N15-H16---O32=C31	0.564–−0.597	1.94	173.69	
SDZ:NVP 1:3	3	N11-H12---O67=C63	0.513–−0.694	1.98	173.39	−27.97
		N12-H13---O50=C46	0.512–−0.629	1.98	174.77	
		N15-H16---O33=C29	0.574–−0.626	1.87	174.87	
SDZ:4-HP 1:5	5	N11-H12---O82-H87	0.486–−0.702	2.09	178.59	−47.91
		N11-H13---O70-H75	0.484–−0.695	2.09	177.57	
		S14=O26---H39-O34	−0.696–0.615	1.78	161.29	
		S14=O27---H51-O46	−0.641–0.604	1.85	172.50	
		N15-H16---O58-H63	0.552–−0.755	1.98	169.17	

**Table 3:** Fitting parameters of the adsorption isotherms for MIPs and NIPs.

Sample	Langmuir			Freundlich		
	*K*_1_ (mL/mg)	*R*_2_	*Q*_m_	*K*_2_ (mL/mg)	*R*_2_	1/*n*
MIPs	10.096	0.9783	27.14	30.025	0.9854	0.4090
NIPs	5.591	0.9413	21.14	18.437	0.8644	0.4098

**Table 4:** Distribution coefficients, selectivity coefficients and relative selectivity coefficients of MIPs and NIPs in the selective adsorption experiment.

	MIPs		NIPs		*K*′
	*K*_d_ (mL/g)	*K*	*K*_d_ (mL/g)	*K*	
SDZ	46.92	—	23.94	—	—
SNM	30.71	1.53	21.42	1.12	1.71
SMX	24.16	1.94	19.97	1.20	2.00
SMM	19.82	2.37	18.49	1.29	2.27

**Table 5:** Adsorption capacity of MIPs in different real water samples.

Real Water Sample	Adsorption Capacity (mg/g)	RSD (%)
Hunhe River	17.08 ± 0.39	2.3
Puhe River	12.73 ± 0.34	2.7
Tap Water	25.36 ± 0.46	1.8

## Data Availability

The raw data supporting the conclusions of this article will be made available by the authors on request.
